# Fabrication of Nb_2_O_5_ Nanosheets for High-rate Lithium Ion Storage Applications

**DOI:** 10.1038/srep08326

**Published:** 2015-02-09

**Authors:** Meinan Liu, Cheng Yan, Yuegang Zhang

**Affiliations:** 1 *i*-Lab, Suzhou Institute of Nano-Tech and Nano-Bionics, Chinese Academy of Sciences, Suzhou, China; 2 School of Chemistry, Physics and Mechanical Engineering, Queensland University of Technology, Brisbane, Australia

## Abstract

Nb_2_O_5_ nanosheets are successfully synthesized through a facile hydrothermal reaction and followed heating treatment in air. The structural characterization reveals that the thickness of these sheets is around 50 nm and the length of sheets is 500 ~ 800 nm. Such a unique two dimensional structure enables the nanosheet electrode with superior performance during the charge-discharge process, such as high specific capacity (~184 mAh·g^−1^) and rate capability. Even at a current density of 1 A·g^−1^, the nanosheet electrode still exhibits a specific capacity of ~90 mAh·g^−1^. These results suggest the Nb_2_O_5_ nanosheet is a promising candidate for high-rate lithium ion storage applications.

Lithium ion batteries are commonly used for consumer electronics, portable electric devices, electric vehicles and other applications^1^,^2^,^3^,^4^,^5^. However, for high power density applications such as in electric vehicles, it is hindered by the achievement of high-rate capability of electrode materials. Recently, transition metal oxides have been extensively explored as anode replacing graphite due to their higher theoretical capacity and high packing densities, which lead to high volumetric energy densities in devices^6^,^7^,^8^,^9^,^10^.

Among the different transition metal oxide as anode materials, lithium titanate (Li_4_Ti_5_O_12_) and niobium oxide (Nb_2_O_5_) have received the most study because of the considerable safety advantage that their redox potentials match to the LUMO of the organic liquid-carbonate electrolyte^11^,^12^,^13^. Compared with Li_4_Ti_5_O_12_ with a specific capacity of 140 mAh·g^−1^, Nb_2_O_5_ exhibits a higher capacity (~200 mAh·g^−1^)^11^,^12^. Furthermore, Augustyn and Dunn *et al* found that the crystal structure of orthorhombic Nb_2_O_5_ permits exceptionally rapid ionic transport since the mostly empty octahedral sites between (001) planes provide natural tunnels for lithium ion transport throughout the *a–b* plane^14^,^15^, which makes Nb_2_O_5_ a promising anode material. However, its intrinsic poor electric conductivity (σ ~ 3 × 10^−6^ S·cm^−1^) and the capacity decay resulted from pulverization during charge-discharge process limit its practical application in lithium ion batteries, and thus it is still challenging to develop efficient but simple ways to enhance the utilization of electroactive Nb_2_O_5_^15^,^16^,^17^,^18^,^19^.

Building nanostructures with desirable morphology and size is of great importance for addressing this issue^15^,^16^,^17^,^18^,^19^,^20^,^21^,^22^,^23^,^24^,^25^,^26^,^27^,^28^,^29^,^30^,^31^. For instance, Dunn *et al* developed Nb_2_O_5_ mesoporous films through a simple solution process, which exhibited high-rate lithium insertion capability^16^. Wang and Lu *et al* reported high performance supercapacitors based on nanocomposites of Nb_2_O_5_ nanocrystals and carbon nanotubes^17^. Nb_2_O_5_-carbon core-shell nanocomposites were fabricated by Li and Ma *et al*, which exhibited high specific capacity and rate capability^18^. Nb_2_O_5_ nanobelts and hollow nanospheres were also been developed with large capacity and high rate capability^19^,^20^.

Among these nanostructures, two dimensional structures with nano size in thickness and micro size in length have been considered to be the appropriate morphology for energy storage^22^,^23^,^24^,^25^. Generally, a nano-sized thickness has short ion diffusion path and large surface area; the micro-sized length can lower the internal resistance and facilitate the electron transfer rate as compared with the noncontinuous oxide framework composed of nanoparticles^22^,^23^,^24^,^25^. These merits encourage us to investigate Nb_2_O_5_ electrode materials with two dimensional structures.

In this work, Nb_2_O_5_ nanosheets were developed by a two-step hydrothermal reaction and subsequent calcination process. The two dimensional sheet-like structure was composed of thin thickness (~50 nm) and long length (~800 nm), fitting the desirable structure principles as mentioned above. As expected, the nanosheet electrodes exhibited superior capacity (~184 mAh·g^−1^), much higher than commercial Nb_2_O_5_ particles (~135 mAh·g^−1^). Additionally, the samples performed well at high current density (~130 mAh·g^−1^ at 0.4 A·g^−1^ and ~90 mAh·g^−1^ at 1 A·g^−1^), indicating its high rate capability. These results demonstrate that our samples are promising for the future practical application in lithium ion storage.

## Results

### Synthesis of intermediate Nb_3_O_7_F nanosheets

**Figure 1:**
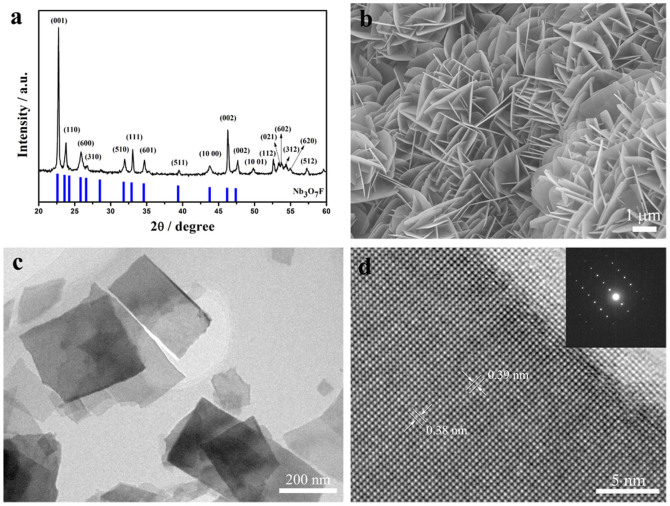
XRD patterns (a) and SEM images (b) of the Nb_3_O_7_F prepared at 160°C with 24 h. The standard diffraction peaks of Nb_3_O_7_F (JCPDF card No 74-2363) are included as reference. TEM image (c) and HRTEM image (d) of the as-obtained Nb_3_O_7_F nanosheets. The inset in (d) is the SAED pattern.

As shown in Fig. 1a, XRD pattern of the as-obtained powders from hydrothermal process can be indexed as an orthorhombic Nb_3_O_7_F structure (JCPDS Card No. 74-2363). Fig. 1b shows the morphology of the as-prepared Nb_3_O_7_F, displaying two dimensional sheet-like structure. A TEM image of Nb_3_O_7_F nanosheets after ultrasound treatment is shown in Fig. 1c. It can be observed that these Nb_3_O_7_F nanosheets are rectangular shape. The nanosheets are almost transparent, suggesting the sheet is very thin. The size of these sheets is around 500 ~ 800 nm. The HRTEM image (Fig. 1d) and SAED pattern (top inset in Fig. 1d) reveal a good single crystalline nature of the Nb_3_O_7_F nanosheets. The lattice fringes show an interplanar spacing of ca. 0.38 nm and 0.39 nm (Fig. 1d), corresponding to the (010) and (001) planes of the orthorhombic Nb_3_O_7_F, respectively.

**Figure 2:**
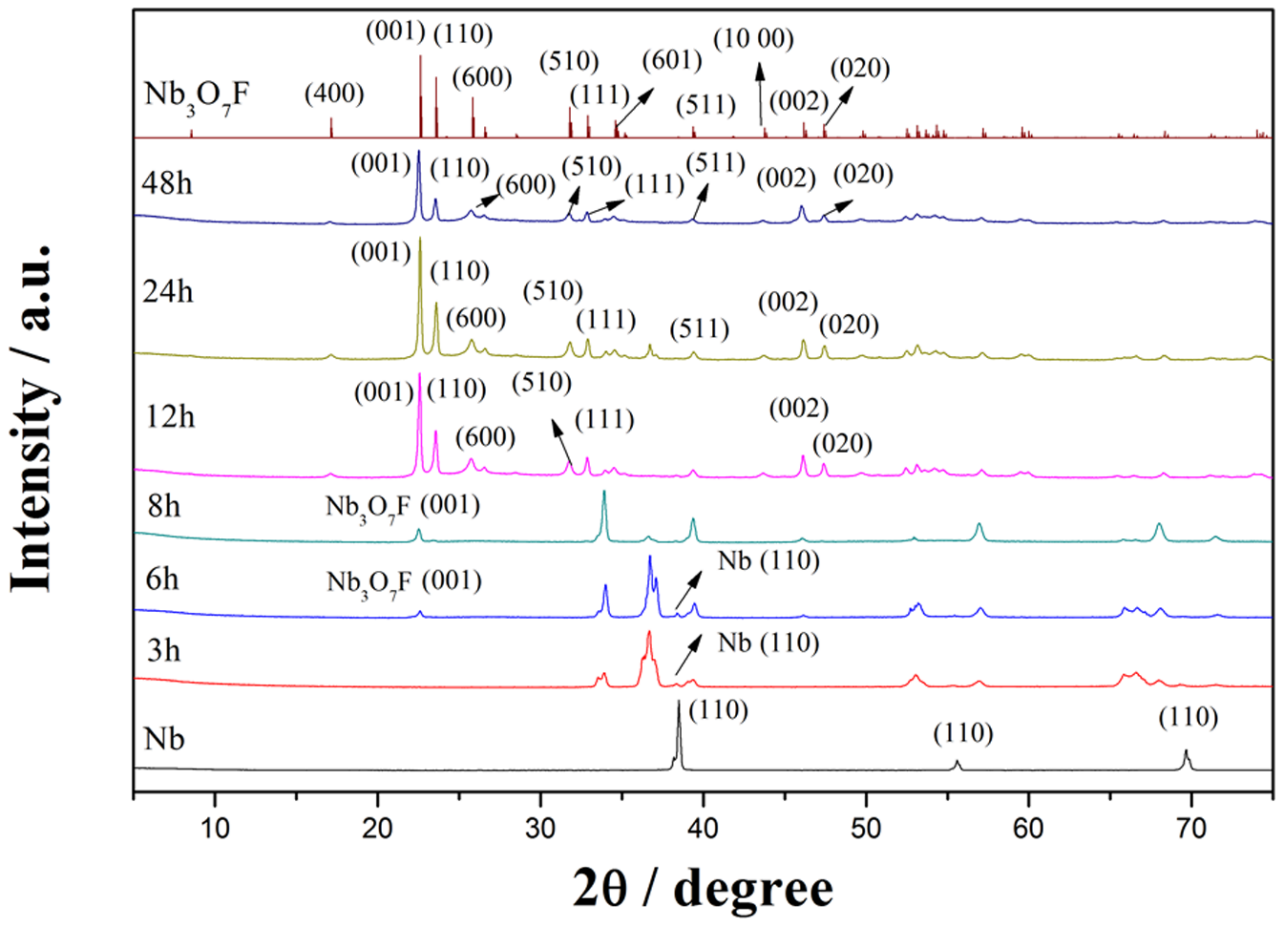
XRD patterns of Nb foil, precursor Nb_3_O_7_F and intermediates obtained with different reaction times. The standard diffraction pattern of Nb_3_O_7_F (JCPDS card No. 74-2363) is shown as a reference.

We investigated the effect of different hydrothermal conditions on the resultant Nb_3_O_7_F crystal structure and morphologies. The reaction time has been found playing a crucial role in controlling the nucleation and growth of crystallites during the hydrothermal system. As shown in Fig. 2, XRD patterns of the solids samples prepared at 160°C with different reaction time indicates pronounced changes in crystal structure during the reaction. XRD patterns of the sample obtained in 3 h are different from that of raw materials Nb, suggesting a new crystal phase forms at the expense of Nb in a short period. With prolonging the reaction time to 6 h, a new diffraction peak appears at 2θ = 22.5°, which can be indexed as Nb_3_O_7_F. Increasing the reaction time to 8 h, it can be found that the diffraction peak at 2θ = 36.6° almost disappear, indicating the crystal phase formed in 3 h degrades readily in the subsequent reaction process. All diffraction peaks of the sample prepared with 12 h are indexed to Nb_3_O_7_F (JCPDS card No. 74-2363). Further prolonging the reaction time to 24 h or 48 h, the XRD patterns are similar, demonstrating the as-obtained samples are pure Nb_3_O_7_F with the reaction time at and longer than 12 h.

**Figure 3:**
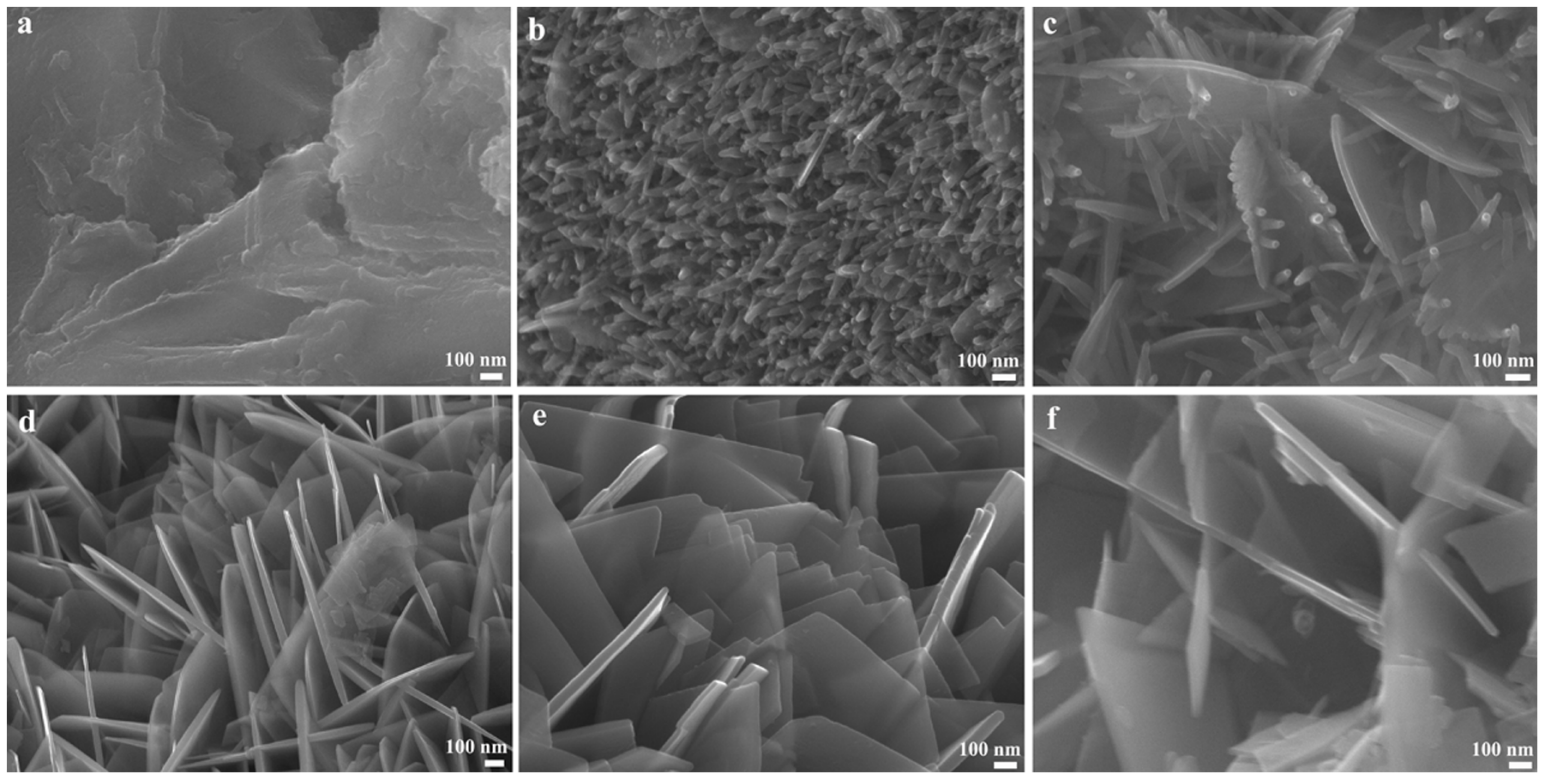
SEM images of the structure evolution of Nb_3_O_7_F nanosheets with increasing times: (a) 3 h; (b) 6 h; (c) 8 h; (d) 12 h; (e) 24 h; (f) 48 h.

The morphology evolution of Nb_3_O_7_F with increasing reaction times was also studied. As shown in Fig. 3a, the samples formed in 3 h are particles. With the reaction time prolonging to 6 h, one dimensional rod-like structure can be observed. The diameter of these rods is around 30 nm (Fig. 3b). Further study indicates that these nanorods appear as a metastable intermediate. For instance, XRD results clearly demonstrate that some new diffraction peaks of Nb_3_O_7_F appear after 8 h of reaction. In addition, this has also been confirmed by SEM observation. With increasing the reaction time to 8 h, some sheets are formed besides those nanorods. The thickness of sheets is around 20 nm and the size is 600 ~ 800 nm. Fig. 3d reveals the presence of a large number of sheets, which indicates that the purity of sheets in the product is increased by prolonging the reaction time. XRD results confirm that the products obtained after reacting 12 h are pure Nb_3_O_7_F. The samples prepared in 24 h are well-crystallized sheets, as shown in Fig. 3e. The thickness of these sheets is around 50 nm, much thicker than that of sheets obtained in 8 h. However, it can be found that most of sheets crack into small pieces with further prolonging the reaction time to 48 h, indicating too long reaction time may damage the perfect sheet-like structure. Therefore, it can be concluded that 24 h is the optimized reaction condition for preparing Nb_3_O_7_F nanosheets.

### Synthesis of Nb_2_O_5_ nanosheets

**Figure 4:**
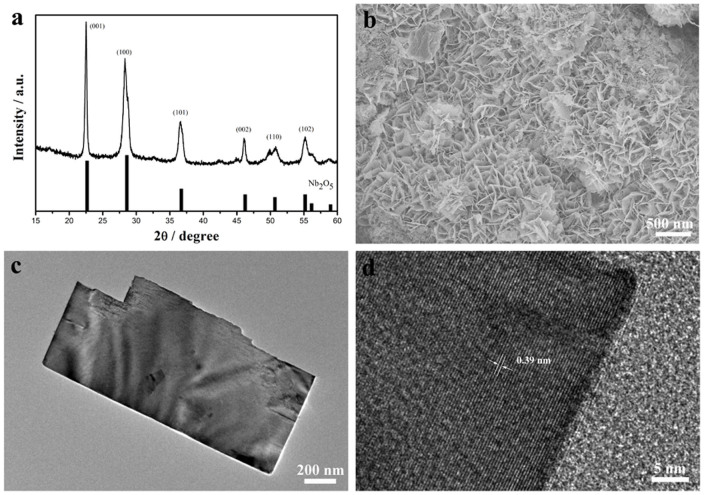
XRD patterns (a) and SEM image (b) of the as-obtained Nb_3_O_7_F nanosheets heated at 550°C in air for 1 h. The standard diffraction pattern of Nb_2_O_5_ (JCPDS card No. 30-0873) is shown as a reference. TEM image (c) and HRTEM image (d) of the as-obtained Nb_2_O_5_ nanosheets.

Ultrathin Nb_2_O_5_ nanosheets could be developed by an in-situ heat treatment of Nb_3_O_7_F nanosheets prepared from optimized reaction condition in air. Fig. 4a shows the XRD pattern of Nb_3_O_7_F heated at 550°C for 1 h. All diffraction peaks are indexed to orthorhombic Nb_2_O_5_ (JCPDS card No. 30-0873). From SEM image in Fig. 4b, it can be observed that the sheet-like structure was kept very well after heating treatment. This is also confirmed by the TEM observation, as shown in Fig. 4c. A typical HRTEM image (Fig. 4d) discloses the lattice fringes with a spacing of 0.39 nm, in a good agreement with the spacing of the (001) planes of Nb_2_O_5_. The results indicate the (001) planes are perpendicular to nanosheets, which may provide natural tunnels for lithium ion transport throughout the *a–b* plane^15^, and thus favour fast intercalation/de-intercalation reaction.

### Electrochemical performance of Nb_2_O_5_ nanosheets

**Figure 5:**
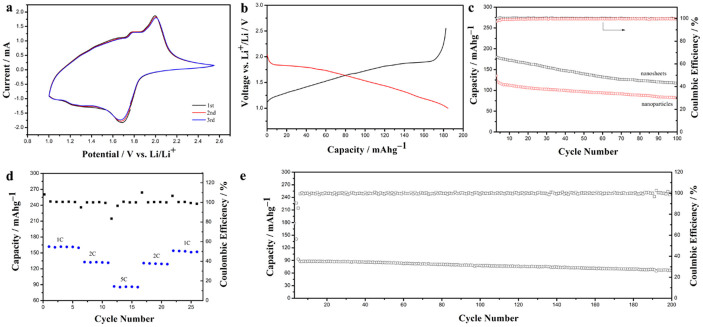
Electrochemical properties of the Nb_2_O_5_ nanosheet electrodes. (a) CV curves of Nb_2_O_5_ nanosheet electrodes; (b) second galvanostatic discharge/charge profiles under 1 C; (c) cycling performance and Coulombic efficiency of the Nb_2_O_5_ nanosheet and nanoparticle electrodes under 1 C; (d) rate performance of the Nb_2_O_5_ nanosheet electrodes; (e) cycling performance and Coulombic efficiency of the Nb_2_O_5_ nanosheet electrodes under 5 C.

High-valent Nb_2_O_5_ is well known to act as electrode material for lithium intercalation (xLi + xe^−^ + Nb_2_O_5_ → Li_x_Nb_2_O_5_). The amount of lithium insertion in Li_x_Nb_2_O_5_ varies between x = 1.6 to a maximum capacity of 200 mAh·g^−1^ (x = 2). Here, the electrochemical properties of lithium ions intercalation (or deintercalation) into (or from) Nb_2_O_5_ nanosheets are characterized. As shown in Fig. 5a, the CV curves of Nb_2_O_5_ at a scan speed of 0.5 mV/s in a potential window of 2.5 to 1.0 V (vs. Li^+^/Li) present symmetric cathodic and anodic peaks, indicating a reversible lithium intercalation and de-intercalation process. Representative voltage profiles at second cycle is shown in Fig. 5b. The curve shows highly reversible capacities.

Fig. 5c compares the charge storage and Coulombic efficiencies of the electrodes from Nb_2_O_5_ nanosheets and commercial Nb_2_O_5_ particles at 0.2 A·g^−1^ (1 C). The initial discharge capacity of this nanosheet electrode is 184 mAh·g^−1^, which almost reaches the theoretical capacity (200 mAh·g^−1^), while the initial discharge capacity of commercial Nb_2_O_5_ particles electrode is only 135 mAh·g^−1^, much lower than that of nanosheet electrode. The capacity can keep 117 mAh·g^−1^ and 83 mAh·g^−1^ after 100 cycles for the nanosheet and particle electrodes, respectively. Kodama *et al* found that the continuous variation in the valence state from Nb^5+^ to Nb^4+^ takes place in the discharge reaction, as xLi + xe^−^ + Nb_2_O_5_ → Li_x_Nb_2_O_5_ (x = 0–2)^21^. This structural variation during the Li intercalation of the two phases could induce strain, which may influence the structure integrity of nanosheets. The structure change of nanosheets could deteriorate the contact between active materials and conducting additives, and thus lead to the capacity fading. The similar phenomenon was also observed in nanobelt electrodes^19^. It should be noted that the Coulombic efficiencies of nearly 100% are achieved for both nanosheet and particle electrodes in this work.

To investigate the rate-capability, the electrode was charged and discharged at 1C to 5C. Fig. 5d shows the rate performance of the nanosheet electrodes. As expected, the specific capacity decreases with the increase of current densities. But the good capacity can be switched back to 2C and 1C again. Furthermore, the nanosheet electrode still exhibits a specific capacity of ~90 mAh·g^−1^ at 5C, indicates the fast reaction kinetics in the electrodes. Fig. 5e shows the cycling performance of the nanosheet electrode at 5C for 200 cycles. It can be found that the specific capacity is around 90 mAh·g^−1^ at the first cycle at 5C and around 70 mAh·g^−1^ after 200 cycles, with Coulombic efficiency stabilized at 100%. The capacity decay rate is as low as 0.11%, which is considered very good for metal oxide nanostructures based electrode materials^20^. The results well demonstrate that Nb_2_O_5_ nanosheets are promising anode materials for high-rate lithium ion storage applications.

## Discussion

### Growth mechanism of intermediate Nb_3_O_7_F nanosheets

Based on XRD and SEM results (in Fig. 2 and Fig. 3), a possible Nb_3_O_7_F nanosheets growth process is presented here. Firstly, Nb powders are slowly etched to H_2_NbF_7_ by HF, and then H_2_NbF_7_ is hydrolyzed into Nb_3_O_7_F. With increasing the hydrothermal reaction time, the Nb_3_O_7_F concentration in reaction solution was further enhanced, which resulted in the formation of Nb_3_O_7_F nanosheets. The chemical reaction process in this hydrothermal system is similar to the results reported by other researchers^32^.

### Improving the electrochemical performance of Nb_2_O_5_ electrodes through constructing 2D microstructures

It is obvious that the specific capacity of Nb_2_O_5_ nanosheet electrode is much higher than that of Nb_2_O_5_ particles, as shown in Fig. 5c. The improved electrochemical performance could be related to the following structural features. First, the micro-sized length (~1 μm) provides a continued pathway for charge transfer, which makes the redox reaction rate faster. Second, the thin thickness (~50 nm) increases the specific surface area, which results in the improvement of the contact area between electrode and electrolyte and facilitated the diffusion of electrolyte into the material, leading to more efficient utilization of the active materials. Third, the (001) planes perpendicular to nanosheets allow degenerate pathways with low energy barriers for ion transport, which may also contribute to the improvement of cycling performance^15^. As shown in Fig. 5e, it can be found that the capacitance retention of 200th cycle is 78% compared to the first cycle at 5C. The nanosheets exhibit superior cycling stability than those nanobelts reported by Wei *et al*, which retains only 72% of the initial discharge capacity after 50 charge/discharge cycles at 0.5C^19^. The superior performance of nanosheets can be attributed to the unique morphology that these sheets not only provide short Li-ion transport length but also accommodate the volume variation. In addition, Sasidharan *et al* reported that their hollow nanospheres also exhibit excellent cycle stability^20^. The capacity retention of 250th cycle is 90% compared to the first cycle^20^. They attribute this excellent cycle performance to the thin-shell of hollow spheres with 6 nm favoring fast intercalation/deintercalation reaction and void space effectively buffering against the local volume changes during repeated charge/discharge processes^20^. As a consequence, the performance of nanosheets could be further improved if the thickness could be reduced in future.

In summary, we have successfully synthesized Nb_2_O_5_ nanosheets from its precursor Nb_3_O_7_F nanosheets. The nanosheet electrode delivers a superior electrochemical performance with an initial discharge capacity of 184 mAh·g^−1^ at 0.2 A·g^−1^ current density. Cycling measurement suggest Nb_2_O_5_ nanosheet electrodes show a high reversible charge/discharge capacity, high rate-capability and excellent cycling stability, making this material a good candidate as an electrode for high-rate electrochemical energy storage applications.

## Methods

### Materials Synthesis

Synthesis of Nb_2_O_5_ nanosheets was performed using the metal Nb powder as the starting materials. In a typical synthesis, 0.15 g Nb metal powder and 0.3 mL HF were added into 30 mL distilled water, then the mixture solution was transferred into a 50 mL Teflon-lined autoclave, and kept it in oven at 160°C for 3 ~ 48 h. The as-prepared precursor was then annealed at 550°C for 1 h in air to obtain Nb_2_O_5_ nanosheets.

### Materials Characterization

The morphologies of precursor and annealed samples were investigated using a field emission scanning electron microscope (FE-SEM, Hitachi S4800) and a field emission transmission electron microscopy (FE-TEM, FEI, Tecnai G2 F20 S-Twin). X-ray diffraction (XRD, Bruker AXS, D8 Advance) was used for crystal structure characterization.

### Electrochemical Measurements

The as-obtained Nb_2_O_5_ nanosheets were mixed with super P and polyvinylidene fluoride (PVDF, Mw = 560 K) in a weight ratio of 80 (active materials):10 (super P):10 (binder), and 1-Methyl-2-pyrrolidinon (NMP) was added to form a homogenous slurry. The slurry was cast onto aluminum current collector using the doctor blade technique. The cast electrode was dried in a vacuum oven at 100°C for 5 h and punched into 15 mm circular discs. CR2016 coin cells were assembled in an Ar-filled glove box with a lithium metal foil as counter electrode and a porous polypropylene separator (2400, Celgard). The electrolyte consisted of a solution of 1 M LiPF_6_ in ethylene carbonate (EC)/dimethyl carbonate (DMC)/diethyl carbonate (DEC) (1:1:1 by volume).

## Author Contributions

M.N.L. designed and conducted the experiment. C.Y. and Y.G.Z. involved in the scientific discussions. M.N.L., C.Y. and Y.G.Z. wrote the manuscript.
